# Role of the 2018 American Thyroid Association statement on postoperative hypoparathyroidism: a 5-year retrospective study

**DOI:** 10.1186/s12893-021-01333-w

**Published:** 2021-09-02

**Authors:** Yuxuan Qiu, Zhichao Xing, Yuan Fei, Yuanfan Qian, Yan Luo, Anping Su

**Affiliations:** 1 Department of Ultrasound, West China Hospital, Sichuan University, No. 37 Guo Xue Xiang, Chengdu, 610041 China; 2 Center of Thyroid and Parathyroid Surgery, Sichuan University West China Hospital, Sichuan Province, No. 37 Guo Xue Xiang, Chengdu, China

**Keywords:** Hypoparathyroidism, Hypocalcemia, Thyroidectomy, American Thyroid Association, Risk factors

## Abstract

**Background:**

Definitions of postoperative hypoparathyroidism (hypoPT) have never reached consent until the American Thyroid Association (ATA) statement was released, with new characteristics and challenges.

**Methods:**

Patients with papillary thyroid carcinoma who underwent primary total thyroidectomy between January 2013 and June 2018 were retrospectively enrolled. Symptoms of hypocalcemia and their frequency were stringently followed. Patients were divided into groups according to the ATA statement. Incidence of postoperative hypoPT and serum parathyroid hormone levels accompanied by calcium levels, from 1-day to at least 24-month follow-up.

**Results:**

A total of 1749 patients were included: 458 (26.2%) had transient and 63 (3.6%) had permanent hypoPT. Transient hypoPT was found in 363 (20.7%) patients with biochemical hypoPT, 72 (4.1%) with clinical hypoPT, and 23 (1.3%) with relative hypoPT; permanent hypoPT was detected in 8 (0.5%) patients with biochemical hypoPT, 55 (3.1%) with clinical hypoPT, and none with relative hypoPT. Female sex, age ≥ 55 years, unintentional parathyroid gland resection, and autotransplantation of ≥ 2 parathyroid glands were independent risk factors for transient biochemical hypoPT. Age ≥ 55 years, bilateral central neck dissection, and isthmus tumor location were independent risk factors for transient clinical hypoPT. A postoperative 1-day percentage of parathyroid hormone (PTH) reduction of > 51.1% was an independent risk factor for relative hypoPT (odds ratio, 4.892; 95% confidence interval, 1.653–14.480; *P* = 0.004). No independent risk factor for permanent hypoPT was found.

**Conclusion:**

ATA diagnostic criteria for postoperative hypoPT are of great value in differentiating patients by hypocalcemia symptoms and choosing corresponding clinical assistance; however, they may underestimate the actual incidence.

## Introduction

In recent years, the global thyroid cancer incidence has risen rapidly, especially for papillary thyroid carcinoma (PTC), which accounts for approximately 85–90% of all thyroid cancers [1]. Thyroidectomy is the primary treatment for PTC, and postoperative hypoparathyroidism (hypoPT) is one of the most common and serious complications. The main causes of hypoPT are as follows: damaged blood supply of the parathyroid gland (PG), which manifests as ischemia or congestion in the PG; direct damage to the PG, including thermal damage or mechanical damage; and accidentally resected PGs. Consequently, insufficient parathyroid hormone (PTH) is produced, and corresponding clinical symptoms are noticed. HypoPT can be divided into transient or permanent hypoPT, and the incidence is approximately 14–60% and 0–33%, respectively [2, 3]. The incidence of hypoPT reported in different studies is quite heterogeneous, and the main reasons for this heterogeneity include different surgical procedures, different experience levels of surgeons, and the use of different diagnostic criteria for hypoPT.

The current definition of hypoPT usually involves the following four indicators: (1) clinical manifestations, that is, whether there are symptoms and/or signs of hypocalcemia; (2) biochemical indicators, that is, serum PTH and/or serum calcium levels; (3) treatment indicators, that is, whether calcium and/or vitamin D treatment is needed to maintain no obvious symptoms and/or signs of hypocalcemia; and (4) duration, that is, the cutoff time that distinguishes transient and permanent hypoPT [4–8]. To solve the problem of complicated and inconsistent diagnostic criteria, the new American Thyroid Association (ATA) statement defines three types of hypoPT: biochemical hypoPT, clinical hypoPT and parathyroid insufficiency (relative hypoPT) [2]. Transient hypoPT is defined as occurring for less than 6 months after surgery, while permanent hypoPT continues beyond 6 months after surgery [2, 9]. The first two definitions are summaries and supplements to previous literature reports, and there are abundant data and studies for reference. Relative hypoPT requires treatment due to symptoms and/or signs of hypocalcemia despite the normal range of serum PTH and calcium levels, which is of some clinical significance.

At present, there is no literature to support the risk factors for hypoPT according to the ATA diagnostic criteria. Therefore, the aim of this comparative study grouped by the above three types of hypoPT is to ascertain the incidence and risk factors for each type and to find the predictors of permanent hypoPT.

## Methods

This study is presented in accordance with the STROBE (Strengthening the Reporting of Observational studies in Epidemiology) checklist [10].

### Patients

We performed a retrospective cohort study including all patients with PTC who underwent primary total thyroidectomy with at least unilateral central neck dissection (CND) at the Center of Thyroid & Parathyroid Surgery, West China Hospital of Sichuan University from January 2013 to June 2018. The exclusion criteria were severe chronic renal insufficiency, preoperative PG dysfunction, reoperation, endoscopic thyroidectomy, lobectomy, completion thyroidectomy, and less than 2 years of follow-up. Neoplasms were grouped into stages according to the American Joint Committee for Cancer (AJCC) staging system (8th edition) [11]. The study was approved by the medical ethics committee of West China Hospital, Sichuan University. The patients involved in the study provided informed consent. All methods were performed in accordance with the relevant guidelines and regulations.

### Indications of total thyroidectomy with lymph node dissection

The indications for total thyroidectomy, instead of lobectomy, were as follows: (1) high-risk radiation exposure or a family history of PTC; (2) bilateral or multifocal PTC; (3) unilateral PTC with contralateral thyroid nodule(s); (4) isthmus PTC; (5) stage T3a PTC > 4 cm; (6) PTC with extrathyroidal extension (ETE) (stages T3b and T4); (7) high-risk pathological variants including tall cell variant, diffuse sclerosis variant, and solid variant; (8) PTC with bilateral central neck lymph node or lateral neck lymph node metastases; (9) PTC with distant metastases; and (10) TERT promoter mutation (confirmed by preoperative fine-needle aspiration biopsy). Unilateral CND was performed routinely unless bilateral CND (BCND) was indicated because of (1) bilateral PTC; (2) isthmus PTC; (3) stage T3 and T4 PTC; (4) prelaryngeal and/or pretracheal lymph node metastases; (5) bilateral central lymph node or lateral lymph node metastases; and (6) TERT promoter mutation (confirmed by preoperative fine-needle aspiration biopsy). Therapeutic lateral neck dissection (LND) (preoperative fine-needle aspiration confirmed) was performed in each patient [4].

### Surgical procedures

All surgeries were performed in our center by four surgeons with similar surgical experience levels and styles. Intraoperative neuromonitoring was used to predict postoperative nerve function [12]. At the same time, carbon nanoparticles were recommended for each patient for better identification of PGs, but their use was finally dependent on the patient’s will. Generally, thyroid or lymph tissues can be stained black by carbon nanoparticles while parathyroid glands cannot because of their different lymphatic drainage. After the first injection of carbon nanoparticles, every attempt was made to identify and carefully preserve each PG and its blood supply to ensure that at least 1 PG was preserved in situ. The resected specimens were examined for any unintentionally removed PGs. When a PG was devascularized or resected unintentionally, selective autotransplantation to the contralateral sternocleidomastoid muscle was performed after confirmation by intraoperative frozen biopsy. The same group of pathologists analyzed all surgical specimens, and the presence of PGs in the surgical specimens was recorded [4, 13].

### Perioperative management

Perioperative management of all patients was standardized. Preoperative examinations included serum calcium, PTH, thyroid function, neck ultrasound, and laryngoscopy. Prophylactic calcium supplementation (calcium gluconate 4000 mg, intravenous drip) was given to each patient on the same postoperative day. If patients had symptomatic hypocalcemia, added and prolonged oral or intravenous calcium supplementation were administered. Serum PTH and calcium levels were also tested 1 day after surgery.

### Follow-up and hypoPT

Laboratory tests, including tests for serum PTH and calcium levels, were routinely performed at 1 month, 3 months, 6 months, 12 months and 2 years after surgery. If necessary, ^131^I ablation was performed by the Department of Nuclear Medicine 2–3 months after surgery. Postoperative hypoPT was defined according to the ATA statement [2]. Biochemical hypoPT was defined as a low intact PTH level, below the lower limit of our center’s standard (1.6–6.9 pmol/L), accompanied by hypocalcemia. Hypocalcemia was defined as a total serum calcium level that was less than the lower limit of our center’s standard (2.1–2.7 mmol/L). Clinical hypoPT was defined as biochemical hypoPT that was accompanied by symptoms and/or signs of hypocalcemia. Relative hypoPT (parathyroid insufficiency) may occur after central neck surgery and typically manifests as clinical symptoms of hypoPT that require medical treatment despite measured laboratory values within normal ranges. Transient hypoPT was defined as occurring for less than 6 months after surgery, while permanent hypoPT continued beyond 6 months after surgery.

### Statistical analysis

All data were analyzed using SPSS 26.0 (SPSS, Chicago, IL, USA) software. Continuous variables (normally distributed) are expressed as mean ± standard deviation (Mean ± SD); variables not normally distributed were expressed as median (range). The t-test (Student's t-test) and one-factor ANOVA test (analysis of variance) were used for continuous variables that conformed to a normal distribution, and the U-test (Mann–Whitney U-test) was used for those that did not. Pearson Chi-square and Fisher's exact tests were used for categorical variables. Independent risk factors were analyzed using binary logistic regression. *P* < 0.05 was considered statistically significant.

## Results

### Population characteristics

**Fig. 1:**
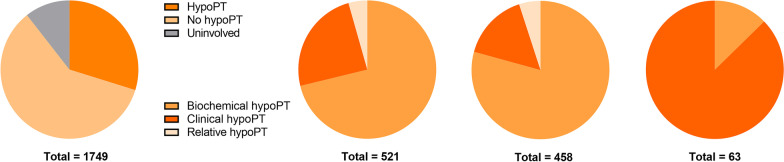
Proportion of each hypoparathyroidism type for the whole cohort, the whole hypoparathyroidism cohort, transient and permanent hypoparathyroidism cohort, respectively

A total of 1928 patients met the inclusion criteria, of whom 179 (9.3%) patients withdrew from the follow-up; 1749 patients were finally included in our analysis. Of the 1749 patients, there were 491 (28.1%) males and 1258 females (71.9%). The overall age was 41.52 ± 11.52 years old, of whom 217 (12.4%) patients were aged ≥ 55 years old. All patients underwent total thyroidectomy, of whom 403 patients (23.0%) underwent unilateral CND and 1346 (77.0%) underwent BCND, including 373 (24.3%) patients who underwent LND. All detailed characteristics are displayed in Table 1. A total of 521 (29.8%) included patients met the ATA diagnostic criteria: 458 (26.2%) patients had transient hypoPT, and 63 (3.6%) patients had permanent hypoPT. Regarding the various types of transient hypoPT, 363 (20.7%) patients had biochemical hypoPT, 72 (4.1%) had clinical hypoPT, and 23 (1.3%) had relative hypoPT. Regarding permanent hypoPT, 8 (0.5%) patients had biochemical hypoPT, 55 (3.1%) had clinical hypoPT, and no patients had relative hypoPT; 1043 (59.6%) patients did not have hypoPT. Approximately 185 (10.6%) patients not only did not meet the ATA diagnostic criteria but were also deemed healthy (e.g., only serum calcium was below the normal range); therefore, they were not included in further data analyses (Fig. 1).

**Table 1:** Population characteristics (N = 1749)

Index	N	%
Age (years)*	41.52 ± 11.52	–
< 55/≥ 55 years	1532/217	87.6/12.4
Male/female	491/1258	28.1/71.9
^131^I ablation	940	53.7
BMI*	23.18 ± 3.91	–
< 24/≥ 24	1126/623	64.4/35.6
Hypertension	144	8.2
Diabetes	46	2.6
Hypothyroidism	26	1.5
Hyperthyroidism	47	2.7
HD	387	22.1
NG	908	51.9
Tumor located at isthmus	85	4.9
Bilaterality	248	14.2
Multifocality	421	24.1
Tumor size (mm)*	13.66 ± 8.94	–
Capsular invasion	366	20.9
Minor ETE	170	9.7
Major ETE	100	5.7
UCND/BCND	403/1346	23.0/77.0
ULND/BLND	328/45	18.8/2.6
Carbon nanoparticles	1287	73.6
Tx/T1a/T1b/T2/T3a/T3b/T4a/T4b	10/652/493/118/241/160/73/2	0.6/37.3/28.2/6.7/13.8/9.1/4.2/0.1
Nx/N0/N1a/N1b	12/857/525/355	0.7/49.0/30.0/20.3
M0/M1	1742/7	99.6/0.4

BMI, Body mass index; HD, Hashimoto’s thyroiditis; NG, Nodular goiter; ETE, extrathyroidal extension; UCND, unilateral central neck dissection; BCND, bilateral central dissection; ULND, unilateral lateral neck dissection; BLND, bilateral lateral neck dissection

*Data presented as mean ± standard deviation

### Biochemical hypoPT

In patients with biochemical hypoPT, 97.8% (363/371) had transient hypoPT, while only 2.2% (8/371) had permanent hypoPT. In univariate analysis, age ≥ 55 years (*P* = 0.016), female sex (*P* = 0.008), BCND (*P* = 0.001), PG unintentional resection (*P* = 0.000), and autotransplantation of ≥ 2 PGs (*P* = 0.000) were associated with transient biochemical hypoPT. In addition, the autotransplantation of ≥ 2 PGs (*P* = 0.025) was associated with permanent biochemical hypoPT (Table 2).

**Table 2:** Clinical characteristics of Biochemical hypoPT

Index	No hypoPT (N = 1043)	Transient hypoPT (N = 363)	Permanent hypoPT (N = 8)	*P1*	*P2*
Age	41.06 ± 11.05	42.67 ± 12.03	46.25 ± 7.87	0.019*	0.403
≥ 55 years	109 (10.5)	55 (15.2)	2 (25.0)	0.016*	0.355
*Gender*					
Male	333 (31.9)	89 (24.5)	1 (12.5)	0.008*	0.685
Female	710 (68.1)	274 (75.5)	7 (87.5)	0.008*	0.685
^131^I	546 (52.3)	205 (56.5)	3 (37.5)	0.175	0.307
BMI	23.23 ± 4.12	23.18 ± 3.84	23.69 ± 2.51	0.838	0.709
> 24	376 (36.6)	125 (35.2)	4 (50.0)	0.645	0.462
Hypertension	84 (8.1)	33 (9.1)	0 (0)	0.538	1
Diabetes	31 (3.0)	9 (2.5)	0 (0)	0.627	1
Hypothyroidism	14 (1.3)	6 (1.7)	0 (0)	0.667	1
Hyperthyroidism	27 (2.6)	13 (3.6)	0 (0)	0.327	1
HD	229 (22.0)	90 (24.8)	1 (12.5)	0.266	0.685
NG	541 (51.9)	193 (53.2)	7 (87.5)	0.670	0.074
Pre PTH	4.96 ± 1.10	4.95 ± 1.07	4.00 ± 1.25	0.878	0.014*
Pre Ca	2.35 ± 0.14	2.36 ± 0.54	2.33 ± 0.15	0.478	0.853
Pre Mg	0.89 ± 0.09	0.91 ± 0.32	0.88 ± 0.04	0.081	0.765
Pre P	1.12 ± 0.20	1.22 ± 1.94	1.13 ± 0.15	0.099	0.895
Pre 25-OH-VD	45.81 ± 17.02	47.91 ± 14.88	56.28 ± 12.51	0.275	0.338
Carbon nanoparticles	787 (75.5)	259 (71.3)	7 (87.5)	0.123	0.450
Unintentionally resected PGs	0.09 ± 0.35	0.19 ± 0.50	0	0.001*	0.000*
≥ 1PG	83 (8.0)	52 (14.3)	0 (0)	0.000*	0.607
Autotransplanted PGs	0.99 ± 0.77	1.24 ± 0.77	0.63 ± 0.52	0.000*	0.025*
No PG	298 (28.6)	66 (18.2)	3 (37.5)	0.000*	0.171
1 PG	474 (45.4)	151 (41.6)	5 (62.5)	0.220	0.289
≥ 2PGs	271 (26.0)	146 (40.2)	0 (0)	0.000*	0.025*
*Neck dissection*					
UCND	277 (26.6)	65 (17.9)	3 (37.5)	0.001*	0.165
BCND	766 (73.4)	298 (82.1)	5 (62.5)	0.001*	0.165
ULND	184 (17.6)	74 (20.4)	1 (12.5)	0.245	1
BLND	21 (2.0)	14 (3.9)	0 (0)	0.052	1
Intraoperative blood loss (mL)	44.25 ± 41.11	58.11 ± 63.77	17.00 ± 4.47	0.000*	0.151
Intraoperative liquid infusion (mL)	1330 ± 700	1500 ± 685	1208 ± 311	0.000*	0.299
*Tumor characteristics*					
Isthmus	48 (4.8)	16 (4.5)	0 (0)	0.826	1
Multifocality	142 (14.1)	52 (14.6)	1 (12.5)	0.830	1
Bilaterality	245 (24.0)	80 (22.2)	1 (12.5)	0.516	1
Capsular invasion	227 (21.8)	66 (18.2)	2 (25.0)	0.148	0.642
Minor ETE	95 (9.1)	44 (12.1)	1 (12.5)	0.098	1
Major ETE	56 (5.4)	26 (7.2)	2 (25.0)	0.209	0.116

BMI, Body mass index; HD, Hashimoto’s thyroiditis; NG, Nodular goiter; Pre, preoperative; PTH, Parathyroid Hormone; Ca, calcium; Mg, Magnesium; P, phosphorus; PG, Parathyroid Gland; UCND, unilateral central neck dissection; BCND, bilateral central dissection; ULND, unilateral lateral neck dissection; BLND, bilateral lateral neck dissection; ETE, extrathyroidal extension

*P < 0.05

In multifactor analysis, female sex (odds ratio (OR), 1.411; 95% confidence interval (CI), 1.067–1.866; *P* = 0.016), age ≥ 55 years (OR 1.503; 95% CI 1.046–2.158; *P* = 0.027), PG unintentional resection (OR 1.703; 95% CI 1.164–2.491; *P* = 0.006) and autotransplantation of ≥ 2 PGs (OR 1.634; 95% CI 1.034–2.580; *P* = 0.035) were independent risk factors for transient biochemical hypoPT. No independent risk factor for permanent biochemical hypoPT was found.

### Clinical hypoPT

In patients with clinical hypoPT, 56.7% (72/127) of patients had transient hypoPT, while 43.3% (55/127) had permanent hypoPT, with a ratio of 1.3:1. A higher incidence of transient clinical hypoPT was found in patients aged ≥ 55 years (*P* = 0.007) and in patients with tumors located at the isthmus (*P* = 0.001), BCND (*P* = 0.001), and autotransplantation of ≥ 2 PGs (*P* = 0.008). Patients with capsular invasion (*P* = 0.032) and intraoperative use of carbon nanoparticles (*P* = 0.015) were less likely to develop transient hypoPT. A lower proportion of permanent clinical hypoPT was found among patients with autotransplantation of ≥ 2 PGs (*P* = 0.048), whereas a higher proportion of clinical hypoPT was found among those with capsular invasion (*P* = 0.034) (Table 3).

**Table 3:** Clinical characteristics of Clinical hypoPT

Index	No hypoPT (N = 1043)	Transient hypoPT (N = 72)	Permanent hypoPT (N = 55)	*P1*	*P2*
Age	41.06 ± 11.05	41.88 ± 13.45	40.89 ± 13.37	0.615	0.683
≥ 55 years	109 (10.5)	15 (20.8)	9 (16.4)	0.007*	0.524
*Gender*					
Male	333 (31.9)	16 (22.2)	13 (23.6)	0.086	0.851
Female	710 (68.1)	56 (77.8)	42 (76.4)	0.086	0.851
^131^I	546 (52.3)	38 (52.8)	36 (65.5)	0.944	0.151
BMI	23.23 ± 4.12	23.29 ± 3.06	23.07 ± 3.88	0.910	0.728
> 24	376 (36.6)	29 (42.0)	18 (32.7)	0.364	0.289
Hypertension	84 (8.1)	6 (8.3)	3 (5.5)	0.933	0.731
Diabetes	31 (3.0)	2 (2.8)	2 (3.6)	1	1
Hypothyroidism	14 (1.3)	1 (1.4)	1 (1.8)	1	1
Hyperthyroidism	27 (2.6)	1 (1.4)	0 (0)	1	1
HD	229 (22.0)	12 (16.7)	15 (27.3)	0.292	0.148
NG	541 (51.9)	31 (43.1)	30 (54.5)	0.148	0.199
Pre PTH	4.96 ± 1.10	4.91 ± 1.19	4.82 ± 1.10	0.698	0.673
Pre Ca	2.35 ± 0.14	2.32 ± 0.10	2.32 ± 0.10	0.042*	0.925
Pre Mg	0.89 ± 0.09	0.91 ± 0.18	0.88 ± 0.08	0.192	0.305
Pre P	1.12 ± 0.20	1.12 ± 0.20	1.14 ± 0.17	0.893	0.508
Pre 25-OH-VD	45.81 ± 17.02	46.79 ± 16.08	48.53 ± 27.66	0.759	0.788
Carbon nanoparticles	787 (75.5)	45 (62.5)	41 (74.5)	0.015*	0.150
Unintentionally resected PGs	0.09 ± 0.35	0.08 ± 0.28	0.13 ± 0.34	0.799	0.422
≥ 1PG	83 (8.0)	6 (8.3)	7 (12.7)	0.909	0.418
Autotransplanted PGs	0.99 ± 0.77	1.29 ± 0.70	1.13 ± 0.70	0.001*	0.191
No PG	298 (28.6)	9 (12.5)	8 (14.5)	0.003*	0.737
1 PG	474 (45.4)	34 (47.2)	34 (61.8)	0.770	0.102
≥ 2PGs	271 (26.0)	29 (40.3)	13 (23.6)	0.008*	0.048*
*Neck dissection*					
UCND	277 (26.6)	6 (8.3)	7 (12.7)	0.001*	0.418
BCND	766 (73.4)	66 (91.7)	48 (87.3)	0.001*	0.418
ULND	184 (17.6)	16 (22.2)	14 (25.5)	0.327	0.671
BLND	21 (2.0)	4 (5.6)	2 (3.6)	0.072	0.697
Intraoperative blood loss (mL)	44.25 ± 41.11	69.49 ± 111.98	62.10 ± 87.73	0.069	0.699
Intraoperative liquid infusion (mL)	1330 ± 700	1526 ± 705	1678 ± 829	0.028*	0.295
*Tumor characteristics*					
Isthmus	48 (4.8)	10 (14.3)	2 (3.6)	0.001*	0.065
Multifocality	142 (14.1)	20 (28.2)	18 (32.7)	0.430	0.696
Bilaterality	245 (24)	11 (15.7)	13 (23.6)	0.716	0.264
Capsular invasion	227 (21.8)	8 (11.1)	14 (25.5)	0.032*	0.034*
Minor ETE	95 (9.1)	7 (55.6)	3 (5.5)	0.863	0.512
Major ETE	56 (5.4)	4 (9.7)	4 (7.2)	0.792	0.726

BMI, Body mass index; HD, Hashimoto’s thyroiditis; NG, Nodular goiter; Pre, preoperative; PTH, Parathyroid Hormone; Ca, calcium; Mg, Magnesium; P, phosphorus; PG, Parathyroid Gland; UCND, unilateral central neck dissection; BCND, bilateral central dissection; ULND, unilateral lateral neck dissection; BLND, bilateral lateral neck dissection; ETE, extrathyroidal extension

*P < 0.05

In multifactor analysis, age ≥ 55 years (OR 2.211; 95% CI 1.150–4.251; *P* = 0.017), BCND (OR 2.878; 95% CI 1.190–6.960; *P* = 0.019), and tumor location at the isthmus (OR 3.473; 95% CI 1.601–7.538; *P* = 0.002) were independent risk factors for transient clinical hypoPT. No independent risk factor for permanent clinical hypoPT was found.

Additionally, patients with clinical hypoPT had a significantly lower PTH level at postoperative day 1 than patients with biochemical hypoPT (0.83 ± 0.28 pmol/L vs 0.93 ± 0.32 pmol/L; *P* = 0.002).

### Relative hypoPT

**Fig. 2:**
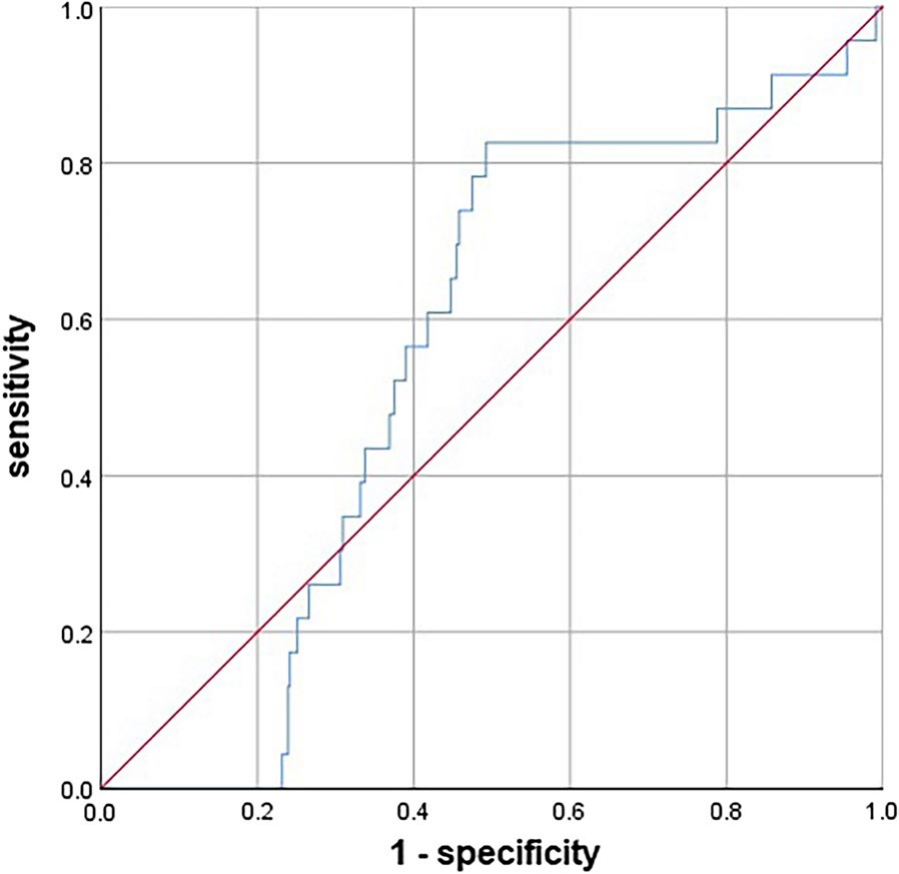
Receiver operating characteristic (ROC) curve for decline rate of parathyroid hormone levels at postoperative 1-day for predicting relative hypothyroidism

Patients with multifocality and bilaterality were more prone to transient relative hypoPT (*P* = 0.032; *P* = 0.014). However, in multivariate analysis, no independent risk factors for relative hypoPT were found (*P* > 0.05; Table 4). To identify additional predictive factors, receiver operating characteristic (ROC) curve analysis was performed (Fig. 2). ROC analysis showed that the percentage of PTH reduction was of some value, and the best cutoff value was 51.1%, whereas the sensitivity was 82.6%, and the specificity was 50.7%. According to univariate analysis, 82.6% (19/23) of the patients with relative hypoPT had a percentage of PTH reduction > 51.1%, while the proportion was only 49.2% (513/1043) in the control group, *P* = 0.002. Logistic regression confirmed that a percentage reduction of PTH > 51.1% at postoperative day 1 significantly increased the risk of relative hypoPT (OR 4.892; 95% CI 1.653–14.480; *P* = 0.004).

**Table 4:** Clinical characteristics of relative hypoPT

Index	No hypoPT (N = 1043)	Transient hypoPT (N = 23)	*P*
Age	41.06 ± 11.05	45.22 ± 9.08	0.073
≥ 55 years	109 (10.5)	3 (13.0)	0.726
*Gender*			
Male	333 (31.9)	6 (26.1)	0.552
Female	710 (68.1)	17 (73.9)	0.552
^131^I	546 (52.3)	16 (69.6)	0.102
BMI	23.23 ± 4.12	24.34 ± 2.81	0.207
> 24	376 (36.6)	12 (54.5)	0.084
Hypertension	84 (8.1)	3 (13.0)	0.426
Diabetes	31 (3.0)	0 (0)	1
Hypothyroidism	14 (1.3)	1 (4.3)	0.281
Hyperthyroidism	27 (2.6)	0 (0)	1
HD	229 (22)	2 (8.7)	0.197
NG	541 (51.9)	14 (60.9)	0.393
Pre PTH	4.96 ± 1.1	5.49 ± 1.03	0.020*
Pre Ca	2.35 ± 0.14	2.33 ± 0.08	0.610
Pre Mg	0.89 ± 0.09	0.95 ± 0.09	0.004*
Pre P	1.12 ± 0.20	1.07 ± 0.15	0.256
Pre 25-OH-VD	45.81 ± 17.02	50.00 ± 15.40	0.467
Carbon nanoparticles	787 (75.5)	18 (78.3)	1
Unintentionally resected PGs	0.09 ± 0.35	0.04 ± 0.21	0.486
≥ 1PG	83 (8.0)	1 (4.3)	1
Autotransplanted PGs	0.99 ± 0.77	1.26 ± 0.75	0.092
No PG	298 (28.6)	3 (13.0)	0.157
1 PG	474 (45.4)	12 (52.2)	0.522
≥ 2PGs	271 (26.0)	8 (34.8)	0.342
*Neck dissection*			
UCND	277 (26.6)	8 (34.8)	0.378
BCND	766 (73.4)	15 (65.2)	0.378
ULND	184 (17.6)	7 (30.4)	0.114
BLND	21 (2.0)	0 (0)	1
Intraoperative blood loss (mL)	44.25 ± 41.11	57.83 ± 63.17	0.316
Intraoperative liquid infusion (mL)	1330 ± 700	1434 ± 895	0.494
*Tumor characteristics*			
Isthmus	48 (4.8)	0 (0)	0.619
Multifocality	142 (14.1)	10 (43.5)	0.032*
Bilaterality	245 (24)	7 (33.3)	0.014*
Capsular invasion	227 (21.8)	2 (8.7)	0.197
Minor ETE	95 (9.1)	4 (17.4)	0.262
Major ETE	56 (5.4)	2 (8.7)	0.360

BMI, Body mass index; HD, Hashimoto’s thyroiditis; NG, Nodular goiter; Pre, preoperative; PTH, Parathyroid Hormone; Ca, calcium; Mg, Magnesium; P, phosphorus; PG, Parathyroid Gland; UCND, unilateral central neck dissection; BCND, bilateral central dissection; ULND, unilateral lateral neck dissection; BLND, bilateral lateral neck dissection; ETE, extrathyroidal extension

*P < 0.05

## Discussion

Many definitions and diagnostic criteria of hypoPT after thyroid surgery are found in the existing literature, leading to widespread controversy over its true incidence. According to a study by Mehanna et al. in 2010, the definition of postoperative hypoPT is almost the most important factor affecting its incidence [14]. The study used 10 different definitions of permanent hypoPT and found that its incidence varied from 0.9 to 4.4% [14]. Furthermore, as postoperative hypoPT began to be noticed, clinicians began an attempt to standardize the diagnostic criteria for postoperative hypoPT. According to a recent systematic review, researchers in 41.6% of studies used hypocalcemia as the definition of permanent hypoPT, and the corresponding incidence was 1.63% (95% CI 0.98–2.42%); researchers in 22.5% of studies used the lower limit of normal serum calcium range as the criterion for permanent hypoPT, and the corresponding incidence was 2.71% (95% CI 1.48–4.37%); and researchers in 36.0% of studies did not describe their definition of permanent hypoPT, and the incidence was 2.97% (95% CI 1.95–4.26%) [6]. As the first study following the ATA statement, we found that the overall incidence of hypoPT was 29.8%, of which the transient hypoPT incidence was 26.2% and the permanent hypoPT incidence was 3.6%. The incidence of postoperative biochemical hypoPT was 21.2%, including 20.7% transient hypoPT and 0.5% permanent hypoPT. The incidence of clinical hypoPT was 7.2%, including 4.1% transient hypoPT, 3.1% permanent hypoPT, and 1.3% relative hypoPT, all of which were transient.

In this study, female sex was the main independent risk factor for postoperative hypoPT. In a study involving 903 patients, 399 (44.4%) of whom were diagnosed with hypoPT, female sex was an independent risk factor for transient hypoPT (OR 1.818; 95% CI 1.326–2.491; *P* < 0.001) but not for permanent hypoPT (*P* = 0.512) [15]. A meta-analysis including 10 studies with 3860 patients who underwent at least total thyroidectomy found that the incidence of postoperative transient hypocalcemia in female patients was significantly higher than that in male patients (OR 1.70; 95% CI 1.03–2.80) [16]. However, the mechanism behind this risk factor for females is not yet clear [7, 17, 18]. There might be differences in the effect of sex hormones on PTH secretion [19]. Different sexes may have different parathyroid tissue monoclonal proliferation and mitotic regulators [20, 21]. Furthermore, there may be differences in the anatomy and morphology of PGs between male and female patients [22, 23]. In addition, studies have shown that this sex difference may be due to the higher rate of vitamin D deficiency in female patients [24].

Patients who underwent total thyroidectomy with CND had a significantly higher incidence of postoperative hypoPT than patients who did not undergo CND. Chisholm et al.'s meta-analysis proved this significantly increased risk, with a risk difference of 7.7 (95% CI 5.6–14.3) [25]. At present, the main issue is whether prophylactic CND should be implemented. Docimo et al. reported symptomatic hypocalcemia developed only in 6.3% of patients, by adopting total thyroidectomy without prophylactic central lymphadenectomy [26]. A large meta-analysis of 23 cohort studies showed that prophylactic CND significantly reduced local recurrence of PTC (OR 0.65; 95% CI 0.48–0.88) but increased postoperative transient hoarseness (OR 2.03; 95% CI 1.32–3.13), transient hypocalcemia (OR 2.23; 95% CI 1.84–2.70) and permanent hypocalcemia (OR 2.22; 95% CI 1.58–3.13) [27]. Compared with unilateral CND, BCND can significantly increase the risk of recurrent laryngeal nerve injury and postoperative hypoPT, but the risk of local recurrence is not improved [28, 29]. Currently, there is agreement between endocrine and neck surgeons about the extension of therapeutic lymph node dissection in N + PTC patients and in the prophylactic treatment of N0 "high risk" patients [30].

In this study, the autotransplantation of only 1 PG had no significant effect on the incidence of transient or permanent hypoPT, while the autotransplantation of ≥ 2 PGs increased the risk for transient hypoPT, but the incidence of permanent hypoPT did not change significantly, even though a preventive trend was detected. Su et al. studied how the number of autotransplanted PGs affected the incidence of postoperative hypoPT. Among the 766 enrolled patients, 36.9% (283 patients) did not undergo PG autotransplantation, and 48.7% (373 patients), 12.7% (97 patients) and 1.7% (13 patients) underwent the autotransplantation of 1, 2, and 3 PGs, respectively. With an increase in the number of autotransplanted PGs of 0, 1, 2, and 3 PGs, the incidence of transient hypoPT was 26.1%, 36.2%, 52.6%, and 84.6%, respectively (*P* < 0.05), and the incidence of permanent hypoPT was 1.8%, 1.1%, 1.0% and 0%, respectively (*P* > 0.05) [4]. Most studies support that intraoperative parathyroid autotransplantation will significantly increase the incidence of transient hypoPT but can effectively decrease the risk of permanent hypoPT. A meta-analysis of 25 original studies found that the autotransplantation of 1 or 2–3 PGs increased the risk of postoperative transient hypoPT (OR 1.71; 95% CI 1.25–2.35; *P* = 0.001; OR 2.22; 95% CI 1.43–3.45; *P* < 0.001), but there was no significant difference in the risk of hypoPT at 6 months after surgery (OR 1.09; 95% CI 0.59–2.01; *P* = 0.781; OR 0.55; 95% CI 0.16–1.87; *P* = 0.341) [16]. Therefore, it is recommended to first emphasize the preservation of both upper PGs in situ and strategic or selective autotransplantation of lower PGs.

The ATA statement chose 6 months as the cutoff time between transient and permanent hypoPT. Our study found that PTH levels recovered between 6 and 12 months after surgery in 16 (25.4%) patients diagnosed with permanent hypoPT, and PTH levels recovered or did not recover after 12 months in 47 (74.6%) patients. From this result, the recovery of parathyroid function within 6 to 12 months is still expected. El-Sharaky et al. believe that most autotransplanted PGs need at least 2–6 weeks to gradually recover their function, but some autotransplanted PGs can take several months to recover [31]. If the blood supply of the PGs preserved in situ is damaged during the operation, the central part of the PGs is affected by avascular necrosis and the formation of new blood vessels for a long time, and functional recovery will be slower [32]. In a study of 854 patients undergoing total thyroidectomy, Villarroya et al. found that a total of 14.5% (142 patients) had postoperative hypoPT, in whom parathyroid function recovered within 6 months in 8.5% (73 patients), recovered within 6–12 months in 2.5% (21 patients), recovered within more than 1 year in 1.4% (12 patients), and did not recover during follow-up in 4.2% (36 patients) [33]. Kim et al. enrolled 1467 patients with total thyroidectomy, with 1.5% (22 patients) diagnosed with permanent hypoPT based on the 6-month time point, but they found that parathyroid function recovered in 0.3% (5 patients) after 6 months [32]. Ritter et al. followed up 1054 patients who underwent total thyroidectomy. A total of 17.9% (189 patients) had hypoPT after surgery, of whom parathyroid function recovered within 2 months in 12.5% (132 patients), recovered between 6 and 12 months in 0.9% (9 patients), and did not recover after 12 months in only 1.9% (20 patients) [34].

In addition to the probable limitation regarding cutoff time, the severity of accompanying hypocalcemia should be taken into consideration. Therefore, we propose the following suggestions about the diagnostic criteria of hypoPT: (1) patients with hypocalcemia (serum calcium level below the lower limit of normal range) needing calcium and/or vitamin D supplementation to relieve the symptoms of hypocalcemia and/or (2) any patient with a serum PTH level lower than the lower limit of normal range. In addition, the cutoff time to distinguish transient and permanent hypoPT should be 1 year. HypoPT should also be divided into mild, moderate, and severe types: mild is defined as hypoPT in which the serum PTH level lower than the lower limit of the normal reference value, with no symptoms of hypocalcemia and no need for calcium and/or vitamin D supplementation (except those who need supplementation due to vitamin D deficiency); moderate is defined as hypoPT in which only oral calcium and/or vitamin D supplementation can relieve the symptoms of hypocalcemia; and severe is defined as hypoPT in which intravenous injection of calcium and/or vitamin D can alleviate the symptoms of hypocalcemia or need of injectable rhPTH (1–84) and other long-acting PTH analogs to keep asymptomatic. In addition, the effects of mental and psychological factors (such as hysteria) should be excluded for moderate and severe cases. These suggestions fully take the impact of symptoms of hypocalcemia on the quality of life of patients into account. Transient symptoms of hypocalcemia that can be relieved by oral calcium supplementation will hardly affect the quality of life of patients. Patients with severe hypoPT need intravenous calcium supplementation to maintain no symptoms of hypocalcemia; therefore, the quality of life of these patients will be greatly affected by the more serious symptoms of hypocalcemia and the necessity to go to a medical institution for injection treatment.

Our study is subject to its retrospective nature and withdrawal rate (9.3%). In addition, there might be some subjective bias of the symptoms of hypocalcemia during follow-up.

## Conclusion

The ATA diagnostic criteria for postoperative hypoPT are of great value in differentiating patients by hypocalcemia symptoms and choosing corresponding clinical assistance, though they may underestimate the actual incidence.

## Data Availability

The data that support the findings of this study are available from the corresponding author upon reasonable request.

## Abbreviations

hypoPT: Hypoparathyroidism
ATA: American Thyroid Association
PTH: Parathyroid hormone
PTC: Papillary thyroid carcinoma
PG: Parathyroid gland
CND: Central neck dissection
ETE: Extrathyroidal extension
BCND: Bilateral central neck dissection
